# SGCP: a spectral self-learning method for clustering genes in co-expression networks

**DOI:** 10.1186/s12859-024-05848-w

**Published:** 2024-07-02

**Authors:** Niloofar Aghaieabiane, Ioannis Koutis

**Affiliations:** Computer Science Department, New Jersey Institute of Technology, Newark, NJ 07102 USA

**Keywords:** Gene co-expression networks, Gene modules, GO enrichment, WGCNA

## Abstract

**Background:**

A widely used approach for extracting information from gene expression data employs the construction of a *gene co-expression network* and the subsequent computational detection of gene clusters, called *modules*. WGCNA and related methods are the de facto standard for module detection. The purpose of this work is to investigate the applicability of more sophisticated algorithms toward the design of an alternative method with enhanced potential for extracting biologically meaningful modules.

**Results:**

We present *self-learning gene clustering pipeline* (SGCP), a spectral method for detecting modules in gene co-expression networks. SGCP incorporates multiple features that differentiate it from previous work, including a novel step that leverages gene ontology (GO) information in a *self-leaning* step. Compared with widely used existing frameworks on 12 real gene expression datasets, we show that SGCP yields modules with higher GO enrichment. Moreover, SGCP assigns highest statistical importance to GO terms that are mostly different from those reported by the baselines.

**Conclusion:**

Existing frameworks for discovering clusters of genes in gene co-expression networks are based on relatively simple algorithmic components. SGCP relies on newer algorithmic techniques that enable the computation of highly enriched modules with distinctive characteristics, thus contributing a novel alternative tool for gene co-expression analysis.

## Background

High throughput gene expression data enables gene functionality understanding in fully systematic frameworks. Gene module detection in Gene Co-expression Networks (GCNs) is a prominent such framework that has generated multiple insights, from unraveling the biological process of plant organisms [1] and essential genes in microalgae [2], to assigning unknown genes to biological functions [3] and recognizing disease mechanisms [4], e.g. for coronary artery disease [5].

GCNs are graph-based models where nodes correspond to genes and the strength of the link between each pair of nodes is a measure of similarity in the expression behavior of the two genes [6]. The goal is to group the genes in a way that those with similar expression pattern fall within the same network cluster, commonly called *module* [7, 8]. GCNs are constructed by applying a similarity measure on the expression measurements of gene pairs. Genes are then clustered using unsupervised graph clustering algorithms. Finally, the modules are analyzed and interpreted for gene functionality [9].

The de facto standard automatic technique for module quality analysis is Gene Ontology (GO) enrichment, a method that reveals if a module of co-expressed genes is enriched for genes that belong to known pathways or functions. Enrichment is a measure of module quality and the module-enriching GO terms can be used to discover biological meaning [9–12]. Statistically, in a given module, this method determines the significance of the GO terms for a test query by associating *p-values*. These are derived based on the number of observed genes in a specific query with the number of genes that might appear in the same query if a selection performed from the same pool was completely random. In effect, these values identify if the GO terms that appear more frequently than would be expected by chance [10]. As usual, the smaller the *p*-value the more significant the GO term.

Several frameworks and algorithms have been developed for GCNs construction and analysis such as [11–17]. Among them, Weighted Correlation Network Analysis (WGCNA) [14], is still the most widely accepted and used framework for module detection in GCNs [5, 9, 11, 12, 18]. WGCNA uses the Pearson correlation of gene expressions to form a ‘provisional’ network and then powers the strength values on its links so that the network conforms with a “scale-freeness” criterion. The final network is constructed by adding to the provisional network additional second-order neighborhood information, in the form of what is called topological overlap measure (TOM). Finally, WGCNA uses a standard hierarchical clustering (HC) algorithm to produce modules [19].

In recent years, there has been a growing interest to enhance WGCNA and multiple frameworks have been proposed as a modification of this framework. These pipelines mainly utilize an additional step in the form of either pre-processing or post-processing to WGCNA. Co-Expression Modules identification Tool (CEMiTool) is a pipeline that incorporates an extra pre-processing step to filter the genes using the inverse gamma distribution [12]. In another study, it is shown that a calibration pre-processing step in raw gene expression data results in increased GO enrichment [9]. Two other frameworks, the popular CoExpNets [11] and K-Module [18], have utilized k-means clustering [20] as a post-processing step to the output of WGCNA. Finally, in a comparative study, CEMiTool appears to have an advantage over WGCNA [21].

All existing frameworks share similar algorithmic components that derive from the original work on WGCNA [14]. Since the inception of WGCNA there has been major progress in algorithms for unsupervised network clustering and their mathematical understanding [22]. This work is informed and motivated by this recent progress. The objective is to adapt and apply these new algorithmic techniques toward the design of an alternative module detection method that can offer a credible and easy-to-use complementary tool for biological discovery.

## Results

We have developed Self-Learning Gene Clustering Pipeline (SGCP), a user-friendly R package for GCNs construction and analysis.^1^ Its integration with Bioconductor makes it easy to incorporate into existing workflows.

### An overview of the SGCP pipeline

**Fig. 1:**
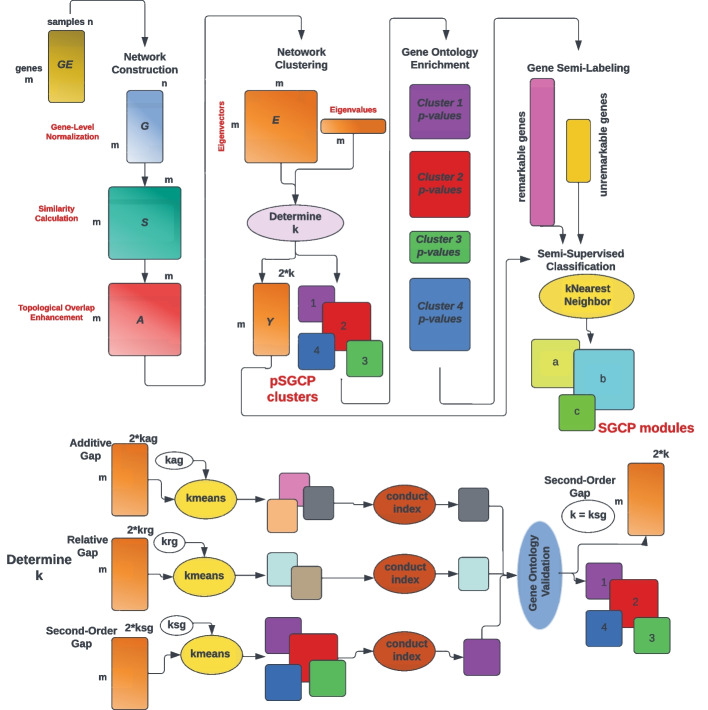
The SGCP pipeline for gene clustering in gene co-expression networks. SGCP takes the gene expression matrix $GE$ and outputs clusters and their refinements to modules after the semi-supervised classification steps. The steps for determining the number of clusters $k$ are drawn below the main pipeline

SGCP differentiates itself from existing frameworks in several ways, discussed in Sect. 3. The workflow of SGCP is illustrated in Fig. 1.

In this subsection, we give an overview of SGCP and also point to the corresponding sections containing more details. SGCP takes as input a gene expression matrix $GE$ with $m$ genes and $n$ sample and performs the following five main steps:

- *Network construction*: Each gene vector, i.e. each row in matrix $GE$ is normalized to a unit vector; this results in a matrix $G$. Next, the Gaussian kernel function is used as the similarity metric to calculate $S$ in which $0 \le s_{i,j} = s_{j,i} \le 1$ and $s_{i,j}$ shows the similarity value between gene $i$ and $j$. Then, the second-order neighborhood information will be added to the network in the form of topological overlap measure (TOM) [13]. The result of this step is an $m\times m$ symmetric adjacency matrix $A$ (Sect. 4.1.1).
- *Network clustering*: Matrix $A$ is used to define and solve an appropriate eigenvalue problem. The eigenvalues are used to determine three potential values $(k_{ag}, k_{rg},k_{sg})$ for the number of clusters *k* (Sect. 4.1.2). For each such value of *k*, SGCP computes a clustering of the network, by applying the kmeans algorithm on an embedding matrix *Y* generated from 2*k* eigenvectors. In each clustering, it finds a *test cluster*, defined as the cluster with the smallest conductance index. The three test clusters are evaluated for GO enrichment, and SGCP picks the clustering that yielded the test cluster with the highest GO enrichment. This clustering is the output of the Network Clustering step, and its clusters are the *initial clusters* (Sect. 4.1.2).
- *Gene ontology enrichment*: GO enrichment analysis is carried out on the initial clusters individually (Sect. 4.1.3).
- *Gene semi-labeling*: Genes are categorized into *remarkable genes* and *unremarkable genes* using information derived from the GO enrichment step. For each cluster, remarkable genes are those that have contributed to GO terms that are more significant relative to a baseline. Remarkable genes are labeled according to their corresponding cluster label. Not all clusters contain remarkable genes, and thus a new number $k' \le k$ of clusters is determined, and accordingly, $k'$ labels are assigned to the remarkable genes and to the corresponding geometric points in the embedding matrix *Y* computed in the Network Clustering step. This defines a supervised classification problem.
- *Supervised classification*: The supervised classification problem is solved with an appropriately selected and configured machine learning algorithm (either k-nearest neighbors [23], or one-vs-rest logistic regression [23]) with the remarkable genes as the training set. The supervised classification algorithm assigns labels to unremarkable genes. At the end of this step, all the genes are fully labeled, and the final clusters called *modules* are produced. SGCP returns two sets of modules, those obtained by the unsupervised Network Clustering step, and those produced by the Semi-supervised classification step. For clarity, in this study, the former and the latter are called *clusters* and *modules* and we denote the corresponding methods with pSGCP (prior to semi-supervised classification) and SGCP respectively (Sect. 4.1.5).

### Comparisons with baselines

We present a summary of our extensive experiments that demonstrate that SGCP outperforms three competing baselines on a wide variety of datasets. The GO enrichment results for all pipelines and all 12 datasets are posted on https://github.com/na396/SGCP.

We compare pSGCP (i.e. SGCP without semi-supervised cluster improvement) and SGCP with three pipelines (WGCNA, CoExpNets, CEMiTool) on 12 gene expression datasets: $4$ DNA-microarray datasets, and $8$ RNA-sequencing datasets as follow. These include expression arrays with a wide range of samples from $5$ to $511$, various organisms, along with different units [24]. Expression units provide a digital measure of the abundance of genes or transcripts. The datasets were downloaded from the NCBI Gene Expression Omnibus (GEO) database [25]. Details on the datasets are available in Table 1. We note that raw DNA-microarray datasets are normalized using robust multiarray analysis (RMA) [26] which is the most popular preprocessing step for Affymetrix [27] expression arrays data [28].

**Table 1:** Benchmark datasets.

Data	Type	Organism	#Samples	Unit	sft
GSE181225 [29]	RNA	Hs	5	RLE	26
GSE33779 [30]	DNA	Dm	90	probes	14
GSE44903 [31]	DNA	Rn	142	probes	30
GSE54456 [32]	RNA	Hs	174	RPKM	30
GSE57148 [33]	RNA	Hs	189	FPKM	14
GSE60571 [34]	RNA	Dm	235	FPKM	9
GSE107559 [35]	RNA	Hs	270	FPKM	3
GSE28435 [36]	DNA	Rn	335	probes	22
GSE104687 [37]	RNA	Hs	377	FPKM	18
GSE150961 [38]	RNA	Hs	418	TMM	5
GSE115828 [39]	RNA	Hs	453	CPM	12
GSE38705 [40]	DNA	Mm	511	probes	16

Possible dataset types are DNA-microarray (DNA) or RNA-seq (RNA). Datasets come from the following organisms: Homo sapiens (Hs), Drosophila melanogaster (Dm), Rattus norvegicus (Rn), Mus musculus (Mm). Units are Relative Log Expression (RLE), Reads Per Kilobase of transcript per Million mapped reads (RPM), Fragments Per Kilobase of exon per Million mapped fragments (FKPM), Trimmed Mean of M-values (TMM). The “sft” column indicates softpower used by tested benchmarks to enforce the network to be scale-free

**Fig. 2:**
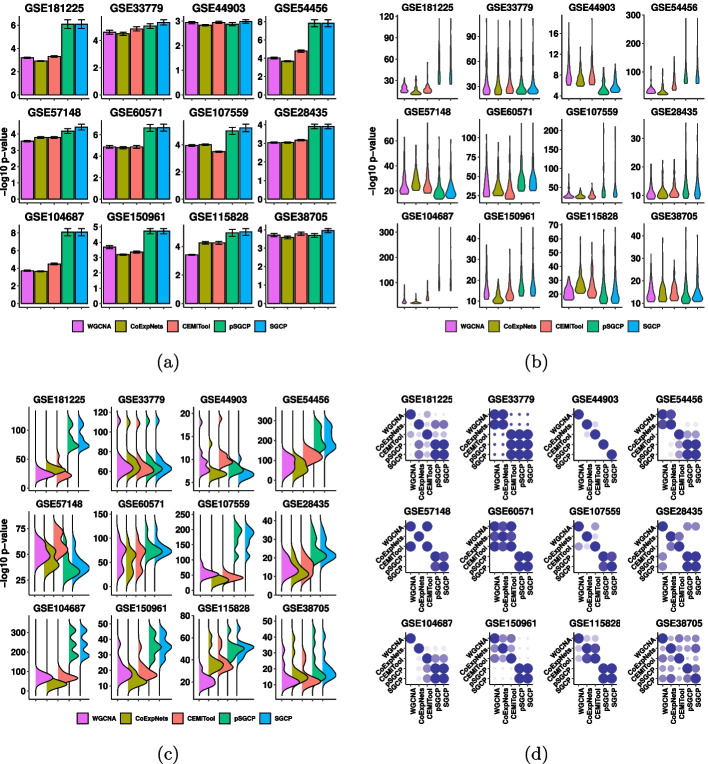
Comparing WGCNA, CoExpNets, CEMiTool, pSGCP, and SGCP for gene ontology enrichment analysis in 12 real datasets: *p*-values are log-transformed. The order of the pipelines from left to right is WGCNA (purple), CoExpNets(yellow), CEMiTool (orange), pSGCP(green), and SGCP (blue). **a** All *p*-values from all modules are pooled, averaged, and shown as a barplot. Error bars indicated the 95% confidence intervals that have been calculated based on the standard deviation of the *p*-values. **b** Top $100$ most significant *p*-values from all modules are shown as a violin plot. **c** Top $10$ most significant *p*-values for the prominent module for each pipeline. **d** Overlaps in top-100 GO terms reported by the five different frameworks. For pipeline *p* in the *x*-axis and pipeline *q* in the *y*-axis, position (*p*, *q*) shows the number of GO terms reported by both *p* and *q*, among their top unique 100 GO terms. The bigger and darker the circle, the higher the overlap

We look at the following metrics of quality.

1. *Average cluster quality.* We follow previous convention and methodology [41, 42], and evaluate performance by comparing the *p*-values returned by pipelines. Let $p_{i,j}$ be the $i$th order *p*-value calculated for module $j$. Then, the quality of module $j$ is defined as $q_j = -(\sum _{j=1}\log _{10} p_{i,j})/n_j$ where $n_j$ is the number of GO terms found in module $j$. Finally, the quality of framework $f$ is defined as $Q_f= (\sum _{i=1}^{k}q_i )/k$ where $k$ is the number of modules in $f$. The results are shown in Fig. 2a. SGCP outperforms the three baselines on all datasets, and the same is true for pSGCP, with the exception of GSE38705. We can also see that SGCP is at least as good as pSGCP on all datasets, and in 6/12 of the datasets, it improves the module quality.
2. *Most significant GO terms.* The summary evaluation includes all *p*-values for the GO terms, as reported by GOstats [43], but here we focus on the top $100$ *p*-values for each pipeline. Figure 2b reports these *p*-values in the form of ‘violin’ plots. The y-axis indicates the significance of each GO term in terms of the *p-value*. The top GO terms in pSGCP and SGCP have a higher *p-value* than the corresponding top terms of the other frameworks except for datasets GSE44903 and GSE57148; in GSE57148 only CEMiTool does better than SGCP. It can be also observed that in $5$ datasets (GSE181225, GSE54456, GSE107559, GSE28435, GSE104687), the *least significant* GO term found by SGCP is more significant than the majority of GO terms founds by the other frameworks. In datasets (GSE150961, GSE11582, GSE60571, and GSE38705) the ‘violin’ for pSGCP and SGCP tends to be higher relative to the other frameworks. In two datasets (GSE33779, GSE38705) the three pipelines have similar performance.
3. *GO terms of most significant module.* We consider as most significant or *prominent*, the module that contains the GO term containing the highest *p*-value. We then consider the 10 most significant GO terms in the prominent module and we show their *p*-values in Fig. 2c. We observe that, even when restricted to the prominent module, pSGCP, and SGCP report more significant terms than other methods, on all datasets except GSE44903 and GSE57148; in GSE57148 only CEMiTool is better than SGCP. In 6 of the datasets, pSGCP and SGCP are astonishingly better than the other frameworks.
4. *Overlap in significant GO terms.* It is interesting to investigate the overlap of GO terms reported by the different frameworks. Here, we focus on the overlapping among the top $100$ GO terms in the prominent module and we show their overlapping in Fig. 2d. The most significant GO terms reported by SGCP are mostly different from those reported by the baseline frameworks. Not surprisingly, pSGCP and SGCP show significant overlaps with each other, as is the case with WGCNA, CEMiTool and CoExpNets, which also share algorithmic components. The overlaps between SGCP and the other three frameworks are smaller, indicating that SGCP reports GO terms that are not reported by the other frameworks.

## Discussion

### Contrasting SGCP with existing frameworks

SGCP deviates from commonly used existing pipelines for GCNs in three key ways:

1. *Network construction:* While existing pipelines employ a procedure that relies on a controversial scale-freeness criterion, SGCP employs a Gaussian kernel whose computation relies on simple statistics of the dataset that are not related to scale-freeness considerations. To the extent that SGCP is effective in practice reveals that scale-freeness is not fundamental in GCNs, affirming the findings of multiple other works on biological networks [44–48].
2. *Unsupervised clustering:* Most existing pipelines employ hierarchical clustering algorithms as the main tool for the unsupervised learning step. SGCP first computes a spectral embedding of the GCN and then applies kmeans clustering on it. Crucially, the embedding algorithm is based on a recent breakthrough in the understanding of spectral embeddings of networks [22].
3. *GO-based supervised improvement:* Existing frameworks do not make any use of GO information, except for providing it in the output. This includes methods that work on improving the quality of a first set of ‘raw’ clusters. SGCP is the first framework that explicitly uses GO information to define a semi-supervised problem which in turn is used to find more enriched modules.

### The effect of supervised re-classification

**Fig. 3:**
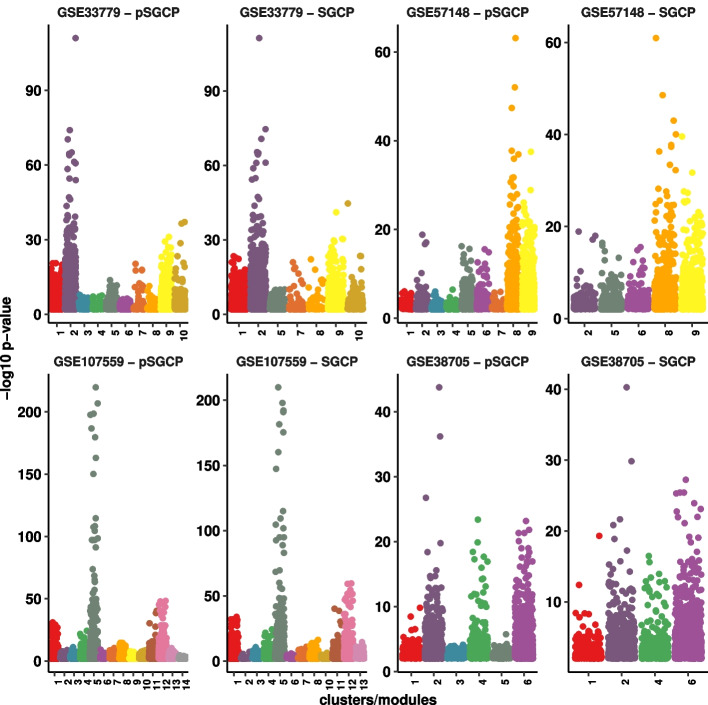
Comparing pSGCP clusters and SGCP modules in $4$ *real datasets.* In all cases, re-classification has resulted in a smaller number of modules relative to clusters. The labels of the eliminated clusters are $3, 4, 6$, in GSE33779, $1, 3, 4, 10$ in GSE57148, $9, 14$ in GSE107559, and $3, 5$ in GSE38705

Once initial clusters are produced, SGCP carries out an additional semi-supervised re-classification of genes to return final modules, as described in Sect. 2. A summary of the impact of this final step is given in Table 2 in the SGCP column. “%UNR Genes” indicates the percentage of the total genes that are *unremarkable*, and “% CH Label” specifies the percentage of unremarkable genes whose label changed after the re-classification. Generally, when the percentage of unremarkable genes is small, the final modules agree with pSGCP clusters; this happens in GSE104687, GSE181225, GSE54456, GSE107559, and GSE150961. In contrast, for a higher percentage of unremarkable genes, SGCP assigns new labels to unremarkable genes and changes significantly the clusters’ shape and size. The highest unremarkable gene percentages occurred in GSE33779, GSE57148, and GSE38705. The difference in enrichment between the clusters (pSGCP) and modules (SGCP) for these data is shown in Fig. 3. It can be seen that, in all cases, the number of clusters gets reduced and the overall enrichment of the modules increases. In GSE107559 the percentage of unremarkable genes is relatively low, but re-classification has wiped out $2$ clusters. In general, if there are clusters that are not enriched the re-classification step eliminates these clusters.

**Table 2:** Summary statistics of applying pipelines WGCNA, CoExpNets, CEMiTool, PSGCP, SGCP

	WGCNA		CoExpNets		CEMiTool		pSGCP		SGCP				
GSE	k	#GO-T	k	#GO-T	k	#GO-T	k	#GO-T	k	#GO-T	mth	% UNR	% CH
181225	48	7462	75	9027	32	6252	2	2598	2	2598	ag,rg	1%	0%
33779	22	5631	19	6213	17	5299	10	4144	7	3821	ag	56%	47.1%
44903	18	3298	27	4705	14	3303	4	987	4	1059	rg	29%	5%
54456	31	9386	46	14473	22	11056	3	6004	3	600	ag,rg	1%	1%
57148	45	13296	36	14110	33	12027	9	2833	5	2383	sg	46%	24%
60571	21	7107	19	8622	16	5564	2	2969	2	2971	ag,rg	21%	2%
107559	26	10915	20	12499	80	15952	14	5257	12	4913	sg	6%	48.4%
28435	51	7331	47	7769	31	6566	2	2052	2	2053	sg	12%	0%
104687	31	10339	28	1193	23	11369	2	6426	2	6426	ag,rg	0%	0%
150961	9	3619	18	606	17	4856	2	2111	2	2111	ag,rg	9%	0%
115828	51	12611	10	7693	10	5231	3	1934	3	1926	sg	33%	0%
38705	8	3320	12	4123	7	3308	6	2824	4	2610	sg	39%	62%

For each of the 5 pipelines, *k* is the number of clusters, and #GO-T indicates the number of gene ontology terms found in all modules collectively by the pipeline; in particular, a single #GO term will appear once for each cluster where its presence exceeds a threshold of significance in term of its *p*-value. In the case of SGCP, “mth” denotes the method ultimately used for selecting $k$, ag: additive gap, rg: relative gap, and sg: second-order gap. %UNR indicates the percentage of the entire genes that are unremarkable. %CH indicates the percentage of the unremarkable genes whose labels have changed after the semi-labeling step

**Fig. 4:**
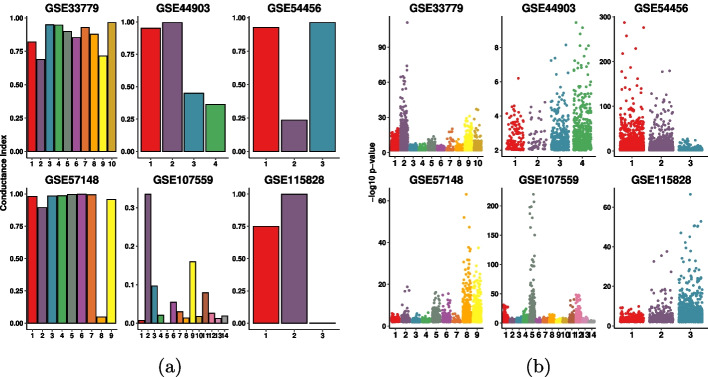
Conductance index and log-transformed *p*-values *analysis in* $6$ *real datasets.* For each data, the conductance index for the clusters (on the left) along with its corresponding log-transformed *p*-values distribution (on the right) is depicted. **a** Conductance index for each module per data. The smaller the bar, the better the cluster. **b** log-transformed *p*-values for each module per data. The higher the point, the more enriched the GO term

**Fig. 5:**
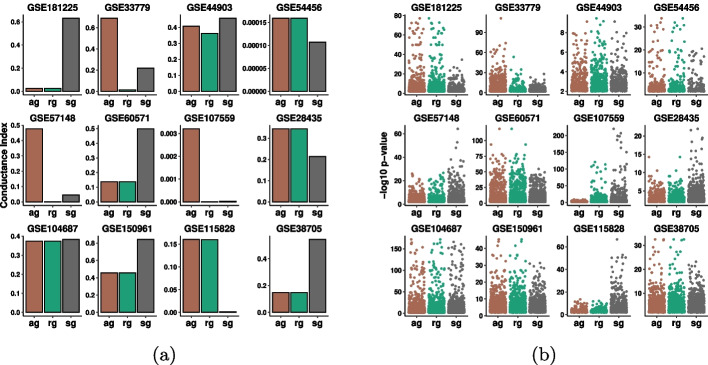
*Conductance index and log-transformed* *p*-values analysis for additive gap (“ag”), relative gap (“rg”), and second-order gap (“sg”) clusters in $12$ *real datasets.* **a** Conductance index for the best cluster of each method on the $12$ datasets. **b** log-transformed *p*-values of the selected clusters for “ag”, “rg”, and “sg” are shown. The higher the point, the more significant the GO term

### The conductance measure

Spectral clustering targets the computation of clusters with a small conductance index as defined in Section 4.1.2 [22]. Thus, when optimizing for conductance, we implicitly hypothesize that smaller conductance should correspond to higher module enrichment. Figure 4 shows this correspondence.

We indeed have observed that there is correspondence between the cluster conductance index and cluster enrichment. Figure 4 shows the conductance index of the modules computed by SGC, along with their corresponding enrichment; here we focus on the cases when $k>2$. It can be seen that in all $6$ data except GSE54456, clusters with smaller conductance indices have higher enrichment. In particular, in GSE107559, the modules with smaller conductance indexes were in order the clusters with label $5, 1, 13, 8, 10, 14, 4, 13$ (see Fig. 4a). Interestingly, from Fig. 4b, it can be seen that these clusters have higher enrichment.

As discussed in Sect. 2, our framework relies on this connection of cluster conductance with enrichment to automatically compute a value of *k* before computing the final clustering and the GO enrichment for the modules. In particular, the method computes the enrichment of three test clusters, that were picked based on their conductance. These clusters’ conductance and enrichment are reported in Fig. 5, where the general correlation between conductance and enrichment is evident.

### SGCP hyperparameters and computation

SPGC requires the computation of a number of eigenvectors. The implementation includes an option that enables the fast iterative computation of the required subset of eigenvectors, thus keeping its runtime to levels comparable with WGCNA and other competing methods. Computing the Gene Ontology Enrichment is a computationally time-expensive task. The process for selecting *k*, described in the Network Clustering step, is meant to reduce the amount of computation for the GO enrichment. However, SGCP enables the user to define their preferred number of clusters *k*. Whenever the amount and time of computation are not of concern, multiple other values of *k* can be evaluated (whenever possible independently, by parallelly running computing processes). This has the potential to produce even better modules. Indeed, in the single case of GSE44903 when our method does not outperform the baselines (see Sect. 2.2), a different choice of *k* does produce a ‘winning’ output for our framework. SGCP also includes a user-defined threshold about the percentage of GO terms used for finding remarkable genes and clusters.

## Methods

### SGCP methods

The input of SGCP is a matrix $GE_{m \times n}$ containing the gene expressions. In GE, rows and columns correspond to genes and samples respectively. Each entry $ge_{i, j}$ is an expression value for gene $i$ in sample $j$. SGCP does not perform any normalization or correction for batch effects and it is assumed that these preprocessing steps have been already performed. SGCP is based on $5$ main steps. Each step offers parameters that can be adjusted by the user.

#### Step I: Network construction

*Gene-level normalization.*

In this step, each gene expression vector, i.e. each row of the matrix $GE_{m \times n}$ is divided by its Euclidean norm which is calculated as

1 $$\begin{aligned} \Vert GE_{i,.} \Vert _{2} = \sqrt{ge^2_{i,1}+ \dots + ge^2_{i,n}}, \end{aligned}$$

where $GE_{i,.} = <ge_{i,1}, \dots , ge_{i,n}>$ is the expression vector of gene *i*. The result of this step is matrix $G_{m \times n}$.

*Similarity calculation.*

We calculate the variance $\gamma ^2$ over all $m^2/2$ pairwise Euclidean distances $\Vert g_{i}-g_{j} \Vert _{2}^2$. We then use $\gamma ^2$ coupled with the following exponential kernel for each pair of genes.

2 $$\begin{aligned} s_{i,j} = k(g_{i},g_{j}) = \exp (\frac{-\Vert g_{i}-g_{j}\Vert _{2}^2}{2 \gamma ^2}). \end{aligned}$$

The result is a similarity matrix $S_{m \times m}$ where $m$ is the number of the genes. Note that $S$ is a symmetric square matrix that ranges from $0$ for the most dissimilar to $1$ for the most similar genes.

*Topological overlap enhancement.*

The adjacency of the network is derived by adding second-order neighborhood information to $S_{m \times m}$ in the form of the topological overlap measure (TOM) [13, 14]. The adjacency strength between gene *i* and *j* is calculated by the following formula:

3 $$\begin{aligned} a_{i,j} = \frac{l_{i,j} + s_{i,j}}{\min {(k_i,k_j)} + 1 - s_{i,j}}, \end{aligned}$$

where $l_{i,j} = \sum _{u}s_{i,u}s_{u,j}$, and $s_{i,j}$ is the similarity coefficient between gene $i$ and $j$ from matrix $S$ of the previous step, and $k_i = \sum _{j}s_{ij}$ is the degree of node *i*. The output is a symmetric adjacency matrix $A_{m \times m}$ with values in $[0,1]$ where $m$ is the number of genes. Note that the diagonal elements of $A$ are zero.

#### Step II: Network clustering

*Eigenvalues and eigenvectors.*

Let $A$ be the adjacency matrix from the previous step. Let *D* be the diagonal matrix containing the degrees of the nodes in the similarity matrix, i.e. $d_{ii} = \sum _{j} a_{ij}$. We perform the following steps:

- Compute the eigenvalues and the corresponding eigenvectors of $D^{-1}A$. Let $\lambda _1,\ldots ,\lambda _m$ be the eigenvalues, and $Y_1,\ldots ,Y_m$ be the corresponding eigenvectors.^2^
- For eigenvector $Y_i$ define the scalar $a_i = 1^T D Y_i/m$, where 1 is the all-ones vector. Then subtract $t_i$ from each entry of $Y_i.$
- Let $$Y_i:= Y_i/(Y_i^T D Y_i)^{1/2}$$.
- Drop the first column of *Y*, as this is a trivial constant vector that does not affect the result.

The output of this step consists of the eigenvalues $\lambda _1,\ldots ,\lambda _m$, and of the matrix of eigenvectors $Y_{m - 1 \times m}$, where eigenvector $Y_i$ is the $i^{th}$ column of *Y*.

*Determining the number of clusters.* Three potentially different values for the number of clusters are calculated, *kag*, *krg*, *ksg* using respectively what we call the *additive gap*, *relative gap*, and the *second-order gap* methods. These are calculated as follows:

4 $$\begin{aligned} kag= & {} {{\,\mathrm{arg\,max}\,}}_i \left( \lambda _{i + 1} - \lambda _{i} \right) \qquad \text {for} \quad i = 2, \dots , m-1 \end{aligned}$$

5 $$\begin{aligned} krg= & {} {{\,\mathrm{arg\,max}\,}}_i \left( \frac{1- \lambda _{i+1}}{1- \lambda _{i}} \right) \qquad \text {for} \quad i = 2, \dots , m-2 \end{aligned}$$

6 $$\begin{aligned} ksg= & {} {{\,\mathrm{arg\,max}\,}}_i \left( \frac{1- \lambda _{i+1}}{1- \lambda _{i}} - \frac{1- \lambda _{i+2}}{1- \lambda _{i+1}}\right) \qquad \text {for} \quad i = 2, \dots , m \end{aligned}$$

The quantities *kag*, *krg*, *ksg* are provisional values for the number of clusters. The final number of clusters is then calculated in the next steps.

*Calculation of conductance index.*

For each of the three possible values of $k$ (i.e. *kag*, *krg*, *ksg*), We set $Y'$ to consist of the $2k$ columns (i.e. eigenvectors) of $Y$. Each row in $Y^{\prime }$ is then divided by its Euclidean norm so that length of each row becomes $1$. Next, the kmeans clustering algorithm [20] is applied on $Y^{\prime }$ to find $k$ clusters using the default kmeans() R function. By default, the maximum number of iterations is set to $10^{8}$ and the number of starts is set to $1000$. Then, for each cluster, the conductance index is computed. Let $C_i$ be one of the clusters. The conductance index for cluster $C_i$ is defined in Eq. 7.

7 $$\begin{aligned} conduct{(C_i)} = \frac{ \sum _{u \in C_i,v \not \in C_i} a_{u,v}}{\sum _{u \in C_i}deg(u)} \end{aligned}$$

where $deg(u) = \sum _{j} A_{u, j}$ which indicates the degree node $u$ (sum of all the weights associated to node $u$), and $a_{u,v}$ is the pairwise association between node $u$ and $v$ in adjacency matrix $A$. For each method, the cluster that has the minimum conductance index is chosen and passed to the next level. Let $c_{ag}$, $c_{rg}$, and $c_{sg}$ denote the clusters with minimum conductance index for the three aforementioned methods respectively.

*Gene ontology validation.*

In this step, the enrichment of clusters $c_{ag}$, $c_{rg}$, and $c_{sg}$ are calculated using the GOstats [43] R package individually for all six possible queries (“underBP”, “overBP”, “underCC”, “overCC”, “underMF”, “overMF‘”) combined. To this end, a conditional “hyperGTest” test is performed and the entire set of genes in the data is considered for the “universeGeneIds”. For each cluster $c \in \{c_{ag}, c_{rg}, c_{sg}\}$, GOstats returns the GO terms found in $c$ along with a *p*-value for each term. Let $P_{i}$ denote the *p-value* associated with a GO term *i* found in $c$. Then the quality of a cluster *c* is determined by:

8 $$\begin{aligned} \sum _{j \in c} -\log _{10}(P_j). \end{aligned}$$

This measure is then used to pick the cluster of best quality among $\{c_{ag}, c_{rg}, c_{sg}\}$. Each of these three clusters was produced by kmeans with a specific choice of *k*: *kag*, *krg*, *ksg* respectively. Then the cluster of best quality directly determines what value of *k* will be used. For example, if $c_{ag}$ is the best cluster, then $k=k{ag}$. After determining *k*, the clusters computed earlier by kmeans for that value of *k* are returned as output, along with embedding matrix $Y^{\prime }_{m \times 2k}$.

#### Step III: Gene ontology enrichment

The GOstats R package [43] is applied to each cluster returned in the GO Validation step. The settings of GOstats are the same as in the GO validation step. GOstats reports answers on user-specified queries including “id”, “term”, “*p*-value”, “odds” “ratio”, “expected count”, “count”, and “size”. SGCP reports this information for each cluster separately. Additionally, for each cluster SGCP reports the GO terms that have been found in the cluster.

#### Step IV: Gene semi-labeling

In the default setting, SGCP picks the top $10 \%$ GO terms according to their associated *p*-values, and consider their corresponding genes as *remarkable*. All other genes are considered *unremarkable*. That percentage is user-adjustable.

With this definition, some clusters may not contain any remarkable genes. Then, each remarkable gene inherits the label of its parent cluster. The unremarkable genes remain unlabeled.

#### Supervised classification

Labeled and unlabeled gene sets along with their corresponding 2*k*-dimensional points given by the rows of $Y^{\prime }$ (obtained in the Network clustering step) define a semi-supervised classification problem. We adopt a simple solution that uses the embeddings of the labeled genes as training points, and we train a simple classifier such as *k-nearest neighbors* (kNN) [49, 50] or logistic regression [51]. Then the trained classifier is used to classify the unlabeled points and their corresponding genes. Note that *k* is the number of clusters determined in the Network Clustering step, but the actual number of clusters returned in this step is equal to the number of clusters found to contain remarkable genes in the *Gene Semi-labeling* step. The default model is kNN and the number of neighbors is ranging from $20:(20 + 2*k)$ if $2*k \le 30$ otherwise $20:30$ depending on accuracy metric using [52] R-package.

### Settings in baseline pipelines

As discussed earlier, all the baseline pipelines use soft-powering (sft) to make the GCNs scale-free. We use the same soft-power methods across all pipelines and the specific powers used for each dataset are reported in Table 1. The functions that are used for GCN construction and analysis in WGCNA, CoExpNets, and CEMiToo, are “blockwiseModules”, “getDownstreamNetwork” and “cemitool” respectively.

## Conclusion

We have proposed SGCP, a novel method for detecting modules of genes in gene co-expression networks. SGCP includes multiple features that differentiate it from existing frameworks and yields modules with significantly higher enrichment in Gene Ontology terms, on multiple benchmark datasets. SGCP identifies clusters whose most significant Gene Ontology terms are markedly different than those identified by WGCNA and other existing frameworks. SGCP’s code is publicly available on Bioconductor, offering an alternative tool for gene co-expression analysis.

## Data Availability

SGCP code, benchmark datasets and all results reported in Sect. 2 can be found in https://github.com/na396/SGCP. SGCP code is also available on Bioconductor (https://bioconductor.org/packages/devel/bioc/html/SGCP.html). The dataset identifiers are as follows: GSE33779 [30], DNA-microarray, provided at https://www.ncbi.nlm.nih.gov/geo/query/acc.cgi?acc=GSE33779. GSE44903 [31], DNA-microarray, provided at https://www.ncbi.nlm.nih.gov/geo/query/acc.cgi?acc=GSE44903. GSE28435 [36], DNA-microarray, provided at https://www.ncbi.nlm.nih.gov/geo/query/acc.cgi?acc=GSE28435. GSE38705 [40], DNA-microarray, provided at  https://www.ncbi.nlm.nih.gov/geo/query/acc.cgi?acc=GSE38705. GSE181225 [29], RNA-sequencing, provided at https://www.ncbi.nlm.nih.gov/geo/query/acc.cgi?acc=GSE181225. GSE54456 [32], RNA-sequencing provided at https://www.ncbi.nlm.nih.gov/geo/query/acc.cgi?acc=GSE54456. GSE57148 [33], RNA-sequencing provided at https://www.ncbi.nlm.nih.gov/geo/query/acc.cgi?acc=GSE57148. GSE60571 [34], RNA-sequencing provided at https://www.ncbi.nlm.nih.gov/geo/query/acc.cgi?acc=GSE60571. GSE107559 [35], RNA-sequencing provided at  https://www.ncbi.nlm.nih.gov/geo/query/acc.cgi?acc=GSE107559. GSE104687 [37], RNA-sequencing provided at https://www.ncbi.nlm.nih.gov/geo/query/acc.cgi?acc=GSE104687. GSE150961  [38], RNA-sequencing provided at https://www.ncbi.nlm.nih.gov/geo/query/acc.cgi?acc=GSE150961. GSE115828 [39], RNA-sequencing provided at  https://www.ncbi.nlm.nih.gov/geo/query/acc.cgi?acc=GSE115828

## Footnotes

1 See https://github.com/na396/SGCP for the latest version.

2 The number of eigenvectors that is practically needed for the rest of the pipeline never exceeds $m'=50$. With an appropriate method, one can calculate at most $m'$ eigenvectors, resulting in a faster method.
